# Characterization of the chicken T cell receptor γ repertoire by high-throughput sequencing

**DOI:** 10.1186/s12864-021-07975-7

**Published:** 2021-09-21

**Authors:** Tongtong Zhang, Qian Li, Xiaoqing Li, Li Kang, Yunliang Jiang, Yi Sun

**Affiliations:** Shandong Provincial Key Laboratory of Animal Biotechnology and Disease Control and Prevention, Shandong Agricultural University, 61 Daizong Street, Shandong Province 271018 Taian City, People’s Republic of China

**Keywords:** Chicken, *TCRγ* locus, High-throughput sequencing, CDR3γ

## Abstract

**Background:**

As one of “γδ-high” species, chicken is an excellent model for the study of γδ T cells in non-mammalian animals. However, a comprehensive characterization of the *TCRγδ* repertoire is still missing in chicken. The objective of this study was to characterize the expressed *TCRγ* repertoire in chicken thymus using high-throughput sequencing.

**Methods:**

In this study, we first obtained the detailed genomic organization of the *TCRγ* locus of chicken based on the latest assembly of the red jungle fowl genome sequences (GRCg6a) and then characterized the *TCRγ* repertoire in the thymus of four chickens by using 5′ Rapid Amplification of cDNA Ends (5′ RACE) along with high-throughput sequencing (HTS).

**Results:**

The chicken *TCRγ* locus contains a single Cγ gene, three functional Jγ segments and 44 Vγ segments that could be classified into six subgroups, each containing six, nineteen, nine, four, three and three members. Dot-plot analysis of the chicken *TCRγ* locus against itself showed that almost all the entire zone containing Vγ segments had arisen through tandem duplication events, and the main homology unit, containing 9 or 10 Vγ gene segments, has tandemly duplicated for four times. For the analysis of chicken *TCRγ* repertoire, more than 100,000 unique Vγ-region nucleotide sequences were obtained from the thymus of each chicken. After alignment to the germline Vγ and Jγ segments identified above, we found that the four chickens had similar repertoire profile of *TCRγ*. In brief, four Vγ segments (including Vγ3.7, Vγ2.13, Vγ1.6 and Vγ1.3) and six Vγ-Jγ pairs (including Vγ3.7-Jγ3, Vγ2.13-Jγ1, Vγ2.13-Jγ3, Vγ1.6-Jγ3, Vγ3.7-Jγ1 and Vγ1.6-Jγ1) were preferentially utilized by all four individuals, and vast majority of the unique CDR3γ sequences encoded 4 to 22 amino acids with mean 12.90 amino acids, which exhibits a wider length distribution and/or a longer mean length than CDR3γ of human, mice and other animal species.

**Conclusions:**

In this study, we present the first in-depth characterization of the *TCRγ* repertoire in chicken thymus. We believe that these data will facilitate the studies of adaptive immunology in birds.

**Supplementary Information:**

The online version contains supplementary material available at 10.1186/s12864-021-07975-7.

## Background

T cells are the central component of the adaptive immune system that is present in all studied jawed vertebrates. The cellular immune system which mediated by T cells visualizes the world of pathogens largely through its T cell immune receptors [1]. Conventional T cell receptors (TCRs) are disulfide-linked heterodimers that are composed by either α and β chains or γ and δ chains, which are co-expressed on the surface of two T cell subpopulations, αβ and γδ T cells, respectively [2]. Each T cell receptor polypeptide is composed of two functional domains: variable region which is capable of recognizing foreign molecular patterns and constant regions which can anchor the receptors in the T cell membrane. During the intrathymic T cell development, the variable region that is unique to each T cell is assembled via somatic recombination of variable (V), diversity (D) and joining (J) gene segments for β and δ chains, and of V and J gene segments for α and γ chains. The antigen specificity of each TCR is largely determined by the complementarity determining region 3 (CDR3) loop, which is encoded by the junctional site of V(D)J rearrangement and is the most varied portion of the TCR molecule [3, 4].

Although γδ T cells represent only a small proportion of the CD3^+^ lymphocytes in the circulation and most tissues in human and mice (“γδ-low” species), they play vital roles as a bridge to connect innate and adaptive immune function. Unlike the antigen recognition by αβ T cells, γδ T cells seem to bind antigens in non-MHC-restricted manners, and the CDR3 length distibutions of the TCRγ and δ indicated that the γδ TCRs may recognize antigen in ways similar to that of antibodies. Therefore, γδ T cells may be more flexible than the classical αβ T cells in mediating cellular immunity [5]. As “γδ-high” species, chicken, rabbit and artiodactyls have high proportion of γδ T cells among circulating lymphocytes. In chickens, the percentage of γδ T cells can reach up to 50 % of isolated lymphocytes of peripheral blood and organs [6]. However, the functions of γδ T cells have not been well studied in these “γδ-high” species. As one of “γδ-high” species and the best-studied non-mammalian model for immunological research, chicken is an excellent candidate for further study of γδ T cells. Elucidating the repertoire diversity of chicken TCR genes will surely provide fundamental information for further understanding the functions of γδ T cells in “γδ-high” species.

At present, the reference germline sequences for the V, D and J gene segments of chicken *TCRγ* locus is not found in the international ImMunoGeneTics information system (IMGT, http://www.imgt.org) [7]. Previous studies reported that the chicken *TCRγ* locus has three Jγ gene segments, a single Cγ gene and three Vγ subgroups, each of which includes approximately 8–10 members [8]. Recently, Liu et al. re-sequenced a bacterial artificial chromosome (BAC) clone 174P24 (~ 205 kb) that covers the red jungle fowl (*Gallus gallus*) *TCRγ* locus by using cross-reference error-correction sequencing approach, Illumina and single-molecule real-time sequencing technology and analyzed the genomic organization of the chicken *TCRγ* locus; however, they did not provide the complete sequence of this BAC clone as well as the detailed germline sequences or locations of each Vγ and Jγ fragments [9].

To obtain a relative complete germline gene database as the basis for downstream repertoire analysis of the chicken *TCRγ*, we focused on the latest assembly of the red jungle fowl genome sequences (GRCg6a, released on Apr 2018, GCA_000002315.5), which was sequenced and assembled with single molecule real time (SMRT) sequencing technology to a depth of approximately 80×. Fortunately, the chromosome region containing *TCRγ* locus possesses few gaps. Therefore, in this study, we first obtained the detailed genomic organization of the red jungle fowl *TCRγ* locus based on these high-quality genome sequences, and then characterized the *TCRγ* repertoire in chicken thymus by using 5′ Rapid Amplification of cDNA Ends (5′ RACE) along with high-throughput sequencing (HTS).

## Methods

### Identification of germline Vγ and Jγ gene segments

Chicken germline Cγ sequence (GenBank accession numbers AB092341) was used as query to retrieve the latest chicken genomic sequences (GRCg6a) by a tBLASTn approach in the GenBank database (www.ncbi.nlm.nih.gov/assembly/GCF_000002315.5/) [10]. To determine the location of the Vγ gene segments, the genomic sequence (~ 100 kb length) located upstream of the Cγ gene was screened using IgBLAST (www.ncbi.nlm.nih.gov/igblast/) [11]. Sequences that matched mouse (or human) Vγ segments with an E-value < 10^− 3^ were further analyzed for chicken Vγ genes.

### Nomenclature of germline Vγ and Jγ gene segments

Since the previous studies have identified three Vγ subgroups [8], in this study we numbered the germline Vγ subgroups according to the previous studies, that is, the Vγ1, Vγ2 and Vγ3 subgroups numbered in this study is one-by-one corresponding to the Vγ1, Vγ2 and Vγ3 subgroups numbered in previous studies. Within each subgroup, Vγ segments are named sequentially in directions from 3′ to 5′ with the subgroup number followed by the gene segment number. Potentially functional, ORF and pseudo-V segments were identified according to the IMGT standards [12]. The V gene domains (framework regions or complementarity-determining regions, FRs or CDRs) were classified using the IMGT numbering system [13]. The alignment and comparison of DNA (and protein) sequences of Vγ segments were performed with DNASTAR lasergene software suite [14] and GeneDoc [15].

### Phylogenetic analyses of germline Vγ gene segments

Phylogenetic tree of Vγ and Jγ segments was constructed in MEGA version X [16] using the maximum likelihood method with 1,000 bootstrap replicates, and phylogenetic trees of chicken Vγ2 segments were constructed using neighbor joining method with 1,000 bootstrap replicates. Only the FR1 through 3 regions (as defined by the IMGT numbering system) of each V sequence were utilized to construct the phylogenetic tree. Multiple nucleotide alignments for the tree construction were performed using ClustalW. Each V subgroup is represented by one sequence per species chosen at random from the functional genes. The accession numbers of V sequences used in this study (except for chicken sequences) are listed in Additional file 1. Chicken sequences were derived from this study.

### Dot plot analyses

Dot plot analyses of red jungle fowl against itself or duck *TCRγ* loci were conducted with dotmatcher (http://emboss.bioinformatics.nl/cgi-bin/emboss/dotmatcher/) [17]. The window size is 300 bp and the identity threshold is 70 %.

### Sample collection, RNA isolation, reverse transcription and quantitative real-time polymerase chain reaction (qRT-PCR)

Four healthy Hy-line Brown commercial hens at the ages of 30 days and 300 days each were purchased from a local chicken farm in Taian city and utilized for isolating total RNA from 13 (30-days-old chicken) or 15 (300-days-old chicken) tissues to analyze the expression pattern of the chicken *TCRγ* gene.

Total RNA was extracted from various tissues using RNAsimple Total RNA Kit (Tiangen Biotech, Beijing, China). Reverse transcription was conducted using PrimeScript RT reagent kit with a gDNA Eraser (TaKaRa, Dalian, China). The mRNA expression level of *TCRγ* was measured by qRT-PCR with primers (CγF and CγR, see Additional file 2) designed according to the mRNA sequence of chicken Cγ segment. The chicken *GAPDH* gene was used as the internal control with primers GAPDHF and GAPDHR (see Additional file 2). qRT-PCR was performed using SYBR Premix Ex Taq (TaKaRa, Dalian, China) on an MX3000p instrument (Stratagene, La Jolla, CA, USA) according to the following conditions: 95 °C for 30 s;40 cycles of 95 °C for 5 s, 53 °C for 30 s, and 72 °C for 15 s; and a final stage 95 °C for 1 min, 58 °C for 30 s, and 95 °C for 30 s. The relative expression levels of a sample were determined using the 2^−ΔΔCt^ method by comparing the values with the internal control. Each sample was amplified in triplicate.

### 5′ rapid amplification of cDNA ends (5′ RACE)

To get the expression diversity of *TCRγ*, total RNA was isolated from the thymus of Hy-line Brown commercial hens at the ages of 30 days using a TRIzol Reagent (Ambion, CA, USA) according to the manufacturer′s instruction. The expressed VJ repertoire of *TCRγ* was obtained by the 5′ RACE method using the SMARTer RACE 5′/3′ Kit (Takara, CA, USA). RACE semi-nested PCR was performed with the forward universal UPM primer and a Cγ-specific reverse primer within the first exon of Cγ (GSP1, see Additional file 2). A unique sequence barcode of 12 nt length was placed at the 5′ end of each GSP1 in order to identify reads that originate from a particular sample (see Additional file 2). All PCR amplifications were performed using two high fidelity enzymes, TransStart FastPfu DNA polymerase (TransGen Biotech, Beijing, China) and PrimeSTAR HS DNA Polymerase (Takara, CA, USA). The detailed protocol for preparation of unbiased TCR cDNA libraries for HTS could refer to the reference [18].

### Library preparation, HTS and data analysis

Library preparation, HTS and data analysis were performed by Beijing Tangtang Tianxia Biotechnology Co., Ltd. Briefly, the 5′ RACE PCR products were detected using agarose gel electrophoresis, and the major DNA bands with the length of 500 ~ 600 bp were recovered and purified. PCR amplicons were then subjected to end-repair and phosphorylation using T4 DNA polymerase, Klenow DNA polymerase and T4 polynucleotide kinase (PNK). These repaired PCR amplicons were 3′ adenylated using Klenow Exo- (3′ to 5′ exo minus, Illumina, CA, USA) and then ligated to the paired-end adapters using T4 DNA ligase (Illumina, CA, USA). Adaptor-ligated products were purified by AMPure XP beads and quantified on an Agilent Technologies 2100 Bioanalyzer. Cluster generation was performed on the cBOT using the TruSeq PE Cluster Kit v3-cBot-HS kit (Illumina, CA, USA) followed by sequencing on Illumina Novaseq 6000 in paired-end mode with a read length of 250 bp.

All of raw reads were treated with a quality control procedure to remove poor quality sequences and adaptor sequences using Cutadapt (version 1.2.1) [19]. Overlapping paired-end reads from the 5′ RACE-based library were merged with FLASH [20], and these merged sequences were aligned to the germline Vγ and Jγ segments identified above using a local BLAST program (version 2.2.30) and each sequence was assigned an optimal germline Vγ and Jγ segments. Those sequences that aligned with a pseudo- or ORF- germline Vγ segment and redundant sequences that have identical CDR3 nucleotide sequence and use the same Vγ and Jγ segments were filtered. According to the IMGT numbering system, the CDR3 of a rearranged TCR gene was defined as the region between the 2nd-conserved cysteine encoded by Vγ region and the Phe-Gly-X-Gly motif encoded by Jγ region [13]. In all potentially functional Vγ segments identified above, the 2nd-conserved cysteine was located in a Tyr(Tyr/His)Cys motif, so DNA sequence between TAC (T/C)A(T/C) TG(T/C) (encoding Tyr(Tyr/His)Cys motif) and TT(C/T) GG(C/A) (A/T)(C/G)(A/T) GG(A/T) (encoding Phe-Gly-X-Gly motif) was extracted from each Vγ-Jγ rearranged sequences using Cutadapt (version 1.2.1) [19]. Putatively non-functional CDR3γ sequences (containing frameshift indels and termination codons) were filtered and the remaining sequences were used to analyze the length distribution and amino acid (AA) composition of the CDR3γ.

### Statistical analysis

Microsoft Excel was used for the HTS data statistics and analysis. Diagrams were conducted using GraphPad Prism version 8.0.2 for windows.

## Results

### Genomic organization of the chicken *TCRγ* locus

**Fig. 1:**
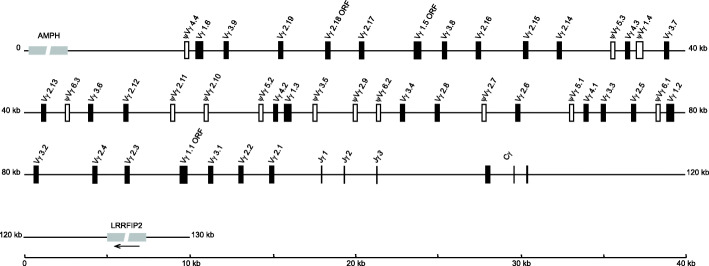
Genomic organization of the red jungle fowl *TCRγ* gene locus. Vγ: potentially functional Vγ gene segment; Vγ ORF: Vγ gene segment with an intact open reading frame but containing defect in RSS or regulatory elements, and/or absence of the conserved amino acids which are necessary for the correct folding of the V-region; Jγ: joining gene segment; Cγ: constant gene segment. Potentially functional Vγ segments and Vγ ORFs are represented with black boxes. Pseudo Vγ segments are represented with hollow boxes and marked with the letter “ψ”. Non-TCR genes located flanking *TCRγ* locus are shown in light grey. The arrow indicates that the transcriptional orientation of *LRRFIP2* gene is opposite to the Cγ gene segment

By annotating the latest assembly of the red jungle fowl genome sequences, we identified the *TCRγ* locus that is mapped on chromosome 2 and spans approximately 100 kb from the most 5′ Vγ gene segment to the 3′ untranslated region (3′ UTR) of the single Cγ region (Fig. 1). The red jungle fowl *TCRγ* locus has a classical translocon organization, similar to opossum (*Monodelphis domestica*), duck, rabbit, Chinese alligator (*Alligator sinensis*) and dolphin (*Tursiops truncate*), but different from human, mouse, Rhesus monkey (*Macaca mulatta*), dromedary (*Camelus dromedarius*), bovine, sheep, cat, dog and Atlantic salmon (*Salmo salar*) [21–34]. As reported previously [8, 35], the locus contains three functional Jγ gene segments with conserved 12-bp RSS at their 5′ end, followed by a single Cγ gene which is encoded by three exons (Fig. 1). A total of 44 Vγ gene segments were identified upstream of the Jγ gene segments. 28 of them are potentially functional; 13 were pseudogenes and three were defined as ORF because of lacking some conserved AA (e.g., 1^st^-CYS 23, TRP 41 and 2^nd^-CYS 104) or RSSs compared with potentially functional Vγ genes (Fig. 1).

**Fig. 2:**
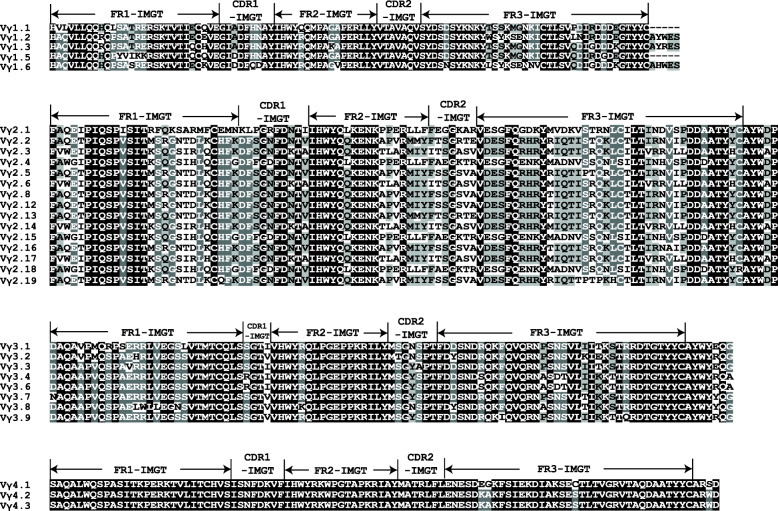
Alignment of amino acid sequences of each potentially functional or ORF Vγ gene segment. The definition of the FRs and CDRs is according to the IMGT unique numbering for the V-REGION

Based on the criterion that the V segments belonging to the same subgroup should share at least 70 % nucleotide identity, 44 Vγ segments could be classified into six distinct subgroups (Table 1). Interestingly, there is no intron between the sequence encoding the leader peptide and the extracellular V domain in the members of Vγ1 subgroup but not in the members of the other five Vγ subgroups. This unusual characteristic is also found in the members of chicken Vα1 subgroup, whereas the V gene segments in chicken *TCRβ* and mammalian *TCRγ* loci exhibit a typical two-exon structure [36, 37]. Subgroup Vγ4, Vγ5 and Vγ6 are only identified in germline sequences but not in cDNA sequences cloned in previous studies [8]. Subgroup Vγ4 contains three potentially functional Vγ segments and one pseudogene, but subgroup Vγ5 and Vγ6 merely contain three pseudogenes, respectively (Table 1). Sequence similarity between the six subgroups showed less than 55 % nucleotide identity (data not shown). There are relatively higher sequences similarity within Vγ1, Vγ3 and Vγ4 subgroups, shared more than 91.1 and 84.0 % identity at the nucleotide and amino acid levels, respectively (data not shown, Fig. 2), but members from Vγ2 subgroup are more diverse than those from other subgroups (Fig. 2 and Additional file 3). Detailed information of each Vγ segment retrieved from the latest chicken genome assembly, including position, transcriptional orientation, nucleotide and amino acid sequence of Vγ, Jγ and Cγ segments, signal peptide sequence, as well as RSS sequence are listed in Additional file 4.

**Table 1:** Summary of the germline Vγ subgroups retrieved from genomic sequences of red jungle fowl (GRCg6a)

Vγ subgroup	Vγ gene	Functional	Total
**Vγ1**	Vγ1.1ORF^a^, Vγ1.2, Vγ1.3, ψVγ1.4^b^, Vγ1.5ORF, Vγ1.6	3	6
**Vγ2**	Vγ2.1, Vγ2.2, Vγ2.3, Vγ2.4, Vγ2.5, Vγ2.6, ψVγ2.7, Vγ2.8, ψVγ2.9, ψVγ2.10, ψVγ2.11, Vγ2.12, Vγ2.13, Vγ2.14, Vγ2.15, Vγ2.16, Vγ2.17, Vγ2.18ORF, Vγ2.19	14	19
**Vγ3**	Vγ3.1, Vγ3.2, Vγ3.3, Vγ3.4, ψVγ3.5, Vγ3.6, Vγ3.7, Vγ3.8, Vγ3.9	8	9
**Vγ4**	Vγ4.1, Vγ4.2, Vγ4.3, ψVγ4.4	3	4
**Vγ5**	ψVγ5.1, ψVγ5.2, ψVγ5.3	0	3
**Vγ6**	ψVγ6.1, ψVγ6.2, ψVγ6.3	0	3
**Total**		**28**	**44**

^a,b^The marks “ORF” and “ψ” are interpreted as Fig. 1

### Phylogenetic analysis of chicken Vγ gene segments

**Fig. 3:**
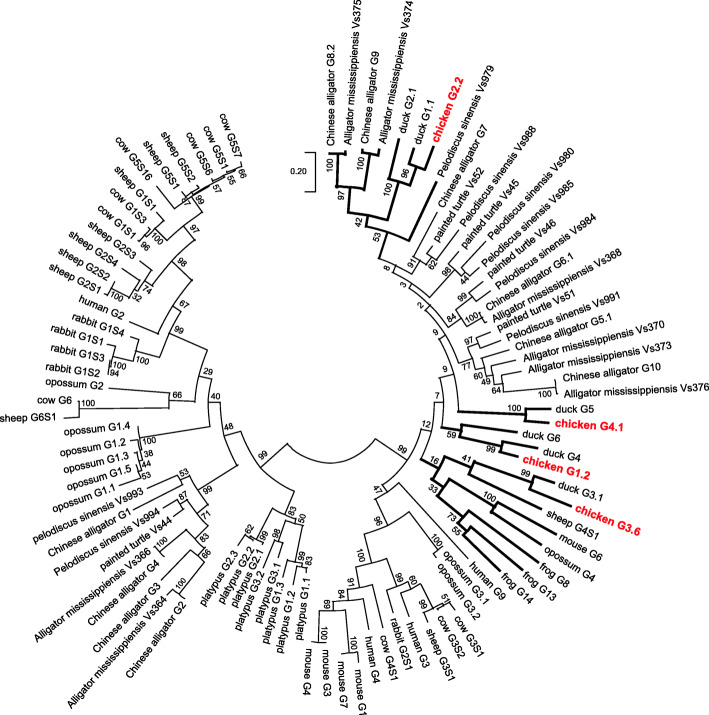
Phylogenetic analysis of the Vγ gene segments in representative tetrapod species. The phylogenetic tree was constructed using the maximum likelihood method in MEGA X with nucleotide sequences corresponding to FR1 through FR3. Branches containing chicken Vγ are indicated in bold, and the chicken Vγ gene segments are marked in red. Bootstrap percentage values based on 1000 replicates are shown at the interior branch nodes. The “G” in the name of each sequence means “Vγ”

The evolutionary relationship of chicken Vγ genes was investigated by constructing a phylogenetic tree with maximum likelihood method using the nucleotide sequences containing FR1 to FR3 region from different tetrapods (Fig. 3). The result shows that there are clear corresponding relationships between chicken and duck Vγ subgroups. The chicken Vγ2 subgroup first clustered with the Vγ1 and Vγ2 subgroups of duck [22], and then clustered with some Vγ subgroups from crocodiles, suggesting that these Vγ subgroups may be evolved from an ancestral Vγ gene that was present in the common ancestor of both birds and crocodiles. Conversely, the chicken Vγ1, Vγ3 and Vγ4 subgroups only clustered with duck Vγ4 (and Vγ6), Vγ3 and Vγ5 subgroups with more than 50 % bootstrap percentage, respectively [22], but clustered with Vγ genes from other tetrapods with lower bootstrap percentage, suggesting that these Vγ subgroups probably emerged after the separation of birds. In general, the phylogenetic analysis of Vγ segments showed that most avian Vγ subgroups have a closer relationship with reptiles rather than mammals. But in some previous studies, chicken Vγ3 first fell in the same phylogenetic clade with Vγ of sheep and cow and then clustered with other mammals, amphibians and/or reptiles [21–23, 38]. The discrepancy between our result and these reports, at least in part, is due to distinct sequences and methods used in phylogenetic tree construction.

### Dot plot analysis of the chicken *TCRγ* locus

**Fig. 4:**
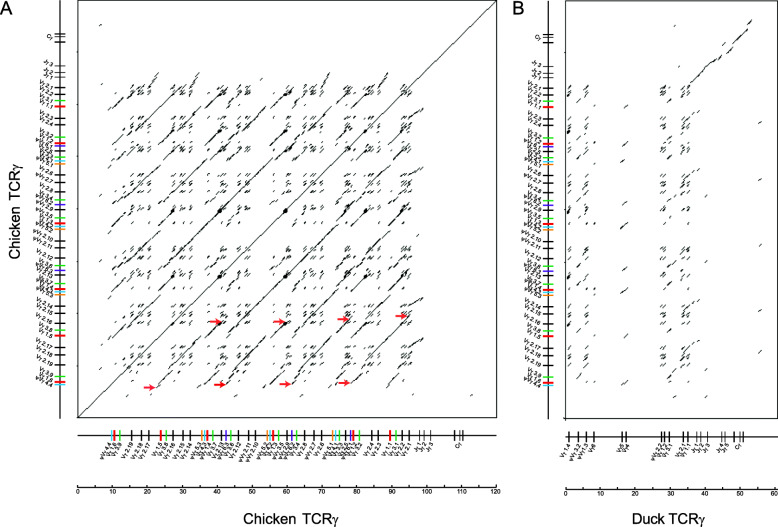
Dot-plot analysis of the chicken *TCRγ* sequence against itself or the duck *TCRγ* sequence. The dot-plot matrices were constructed using dotmatcher with 300 bp window size and 70 % identity threshold. Dots and lines indicate the regions that showed more than 70 % nucleotide similarity within the chicken *TCRγ* locus (**A**) or between chicken and duck *TCRγ* loci (**B**). In matrix A, the main homology units are indicated with red arrows. Different Vγ subgroups are shown in different colors in the schematic representation of chicken *TCRγ* locus alongside axes. Vγ segments from Vγ1 to Vγ6 subgroups are depicted in red, black, green, blue, yellow and purple, respectively

To clarity the genomic structure and possible evolution mode of the chicken *TCRγ* locus, the red jungle fowl *TCRγ* genomic sequence was aligned against itself by dot-plot analysis (Fig. 4A). The dot-plot matrix clearly shows that a series of tandem duplication events had led to a substantial increase in the number of germline Vγ genes. The main homology unit, containing 9 or 10 Vγ gene segments, has tandemly duplicated for four times, which covers almost the entire zone of Vγ genes. All four repeats are nearly identical in length (16 ~ 18 kb) and share more than 83.3 % nucleotide identity (see Additional file 5), suggesting that they might be produced by recent duplication events. Our previous study showed that the 5′ part of the chicken *TCRβ* locus also generated from tandem duplication occurred recently [37], so tandem duplication may be a common mechanism used to construct the TCR loci in chicken.

In the dot-plot matrix obtained from the comparison between the red jungle fowl and duck *TCRγ* loci (Fig. 4B), we can clearly find that there was no region longer than 5 kb with high level of pairwise identity in the 5′ part of the *TCRγ* loci between chicken and duck, but the Jγ-Cγ regions of chicken and duck show higher nucleotide identity, indicating that the Jγ-Cγ region remains conserved in *Anas* and *Gallus* during birds evolution. However, this homology portion is interrupted due to insertion of a fragment containing Jγ4 and Jγ5 segments in duck. Phylogenetic analysis of the chicken and duck Jγ segments shows that the chicken Jγ1, Jγ2 and Jγ3 segments are tightly clustered with the duck Jγ1, Jγ2 and Jγ3 segments, respectively (see Additional file 6), but the duck Jγ4 and Jγ5 segments which have nearly identical nucleotide sequences seem to have no corresponding Jγ segment in chicken but are clustered with chicken/duck Jγ3 with a relatively low bootstrap percentage (53 %), suggesting that either Jγ4 or Jγ5 might first evolve from a duplication of the Jγ3 occurred earlier after the speciation of *Anas* and *Gallus*, and this Jγ duplicated again to form current Jγ4 and Jγ5 segments in duck *TCRγ* locus.

### Expression of chicken *TCRγ* gene in various tissues

**Fig. 5:**
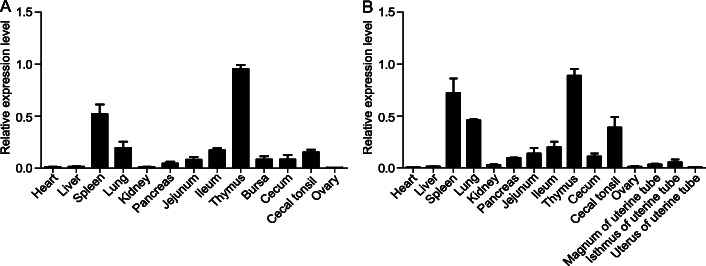
The relative expression levels of *TCRγ* gene in different tissues. qRT-PCR analysis of the relative expression levels of *TCRγ* gene in different tissues of Hy-line Brown hens aged 30 days (**A**) and 300 days (**B**). The chicken *GAPDH* gene was selected as an internal control. The vertical axis indicates the normalized fold changes in expression calculated using the 2^−ΔΔCt^ method, and the tissues are listed below the horizontal axis. Data are representative of four independent samples, and the error bar represents the standard deviation of the mean

The expression profile of chicken *TCRγ* genes in different tissues, which were sampled from Hy-line Brown hens at the ages of 30 days and 300 days, was assessed by qRT-PCR. In 30-days-old chickens (Fig. 5A), *TCRγ* was highly expressed in the thymus and spleen, and relatively weakly in the lung and gut. In 300-days-old chickens (Fig. 5B), *TCRγ* was also highly expressed in the thymus and spleen, and the expression in the lung and gut seemed to be higher than that in the 30-days-old chickens. The relatively lower expression of *TCRγ* in gut may probably be attributed to the tissue for RNA extraction is the gut wall but not the gut epithelium where the chicken γδ T cells are mainly found [39]. Unexpectedly, in 300-days-old laying hens, *TCRγ* was still expressed at the highest level in the thymus. The *TCRγ* expression level did not decrease due to thymic degeneration as expected, and the reason of this phenomenon need to be further explored.

### Diversity of *TCRγ* transcripts in chicken thymus

**Fig. 6:**
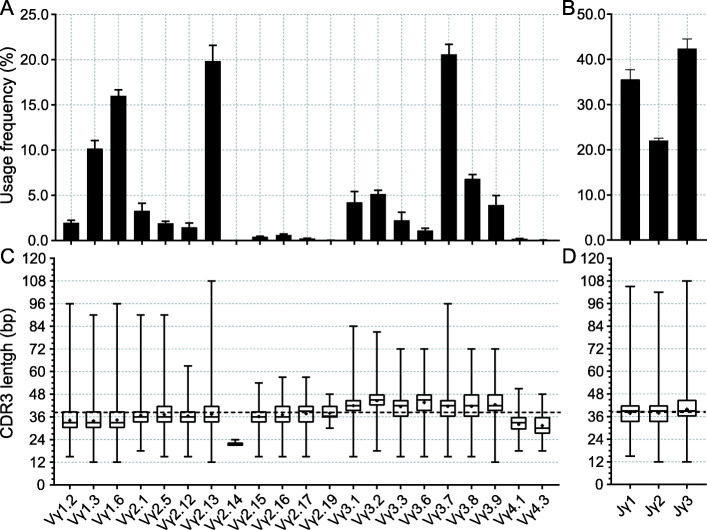
Usage frequency of each Vγ and Jγ segment and their corresponding mean CDR3γ length. The usage frequencies of each Vγ and Jγ segment are shown in figure **A** and **B**, and the mean nucleotide length of CDR3γ corresponding to each Vγ and Jγ segment are shown in figure **C** and **D**. In figure **C** and **D**, the boxplots represent the nucleotide length distribution of CDR3γ (including the sum of all functional CDR3γ sequences from four individuals) for each Vγ and Jγ segment. The upper and lower ends of a rectangular box represent the third quartile and first quartile of the CDR3γ length, respectively. The horizontal line and the plus sign inside the box indicate the median and the mean of the CDR3γ length, respectively. The black dotted line represents the mean length of CDR3γ (38.68 bp) calculated from all functional CDR3γ sequences

**Fig. 7:**
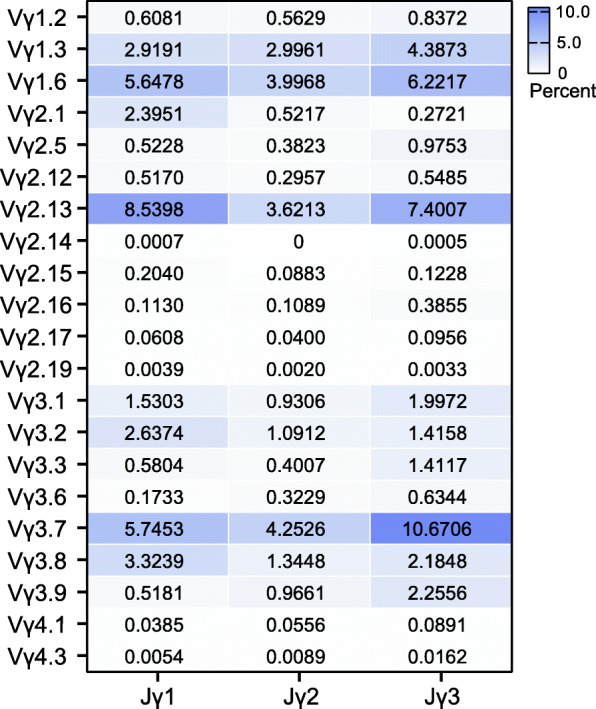
Usage frequencies of all possible Vγ-Jγ pairs. The vertical axis represents all potentially functional Vγ segments and the horizontal axis represents three Jγ segments. The color depth is proportional to the usage frequency of a certain Vγ-Jγ pair

Based on 5′ RACE assay and HTS, we analyzed the *TCRγ* repertoire from thymus of four 30-days old Hy-line Brown hen. A total of 200,114, 121,916, 105,939 and 170,315 unique V-region nucleotide sequences were obtained from four samples, respectively. By alignment of each unique V-region sequence with the germline Vγ and Jγ sequences identified in red jungle fowl *TCRγ* locus, 369, 222, 235 and 324 sequences that utilized pseudo- or ORF- germline Vγ segments were filtered from four samples, respectively, and the rest *TCRγ* transcripts (199,745, 121,694, 105,704 and 169,991 sequences) were analyzed the combinational diversity. In general, all four Vγ subgroups containing potentially functional Vγ segments participated in Vγ-Jγ rearrangement (Fig. 6A). Members of Vγ3 subgroup (43.98 %) appeared to be more frequently utilized than those of Vγ1 (28.12 %) and Vγ2 (27.68 %) subgroups (data not shown). There was also a usage preference of several Vγ segments, including Vγ3.7, Vγ2.13, Vγ1.6 and Vγ1.3, which account for 20.58 %, 19.84 %, 16.00 and 10.15 % of the expressed Vγ repertoire, respectively. The two functional members of Vγ4 subgroup, Vγ4.1 and Vγ4.3, took part in V-J rearrangement but with very low frequencies (0.19 and 0.03 %), which is probably the reason why this subgroup has not been identified by traditional cloning and sequencing methods. All three Jγ gene segments were utilized in Vγ-Jγ rearrangement, with a little biased usage of Jγ3 segment (42.38 %) compared with Jγ1 (35.58 %) and Jγ2 (22.04 %) segments (Fig. 6B). Combinations of the above dominantly expressed Vγ and Jγ segments formed several favoured Vγ-Jγ pairs (Fig. 7). The Vγ3.7-Jγ3 was most frequently used pair with 10.67 % percent in all combinations, and the top six pairs, including Vγ2.13-Jγ1, Vγ2.13-Jγ3, Vγ1.6-Jγ3, Vγ3.7-Jγ1 and Vγ1.6-Jγ1, totally accounted for more than 44 % of the entire repertoire. No dominantly deviation was observed in comparison of preferred Vγ-Jγ pairs between individuals (see Additional file 7).

**Fig. 8:**
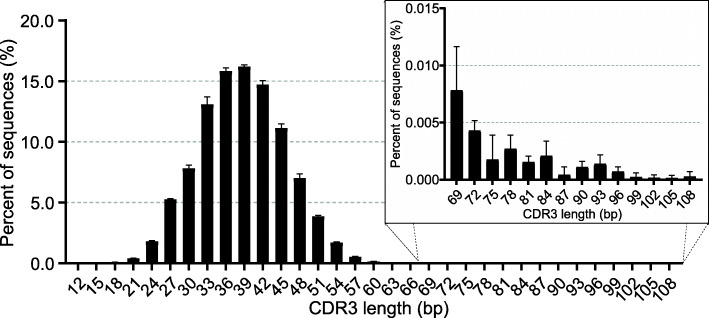
Length distribution of CDR3γ. The nucleotides encoding the 2nd-conserved cysteine and the conserved phenylalanine are not included in the CDR3γ. The horizontal axis represents the nucleotide length of CDR3γ region. The bar height indicates the mean frequency from four individuals, and the error bars show standard deviations

To identify the junctional diversity of the rearranged *TCRγ* transcripts, we first filtered the sequences containing putatively non-functional CDR3 region. After filtering, 183,476, 112,860, 97,203 and 156,224 sequences containing functional CDR3 region were retained from four samples, respectively, which account for 91.86 %~92.74 % of the unique V-region sequences rearranged from functional Vγ and Jγ (data not shown). These sequences were used to analyze the length distribution and AA composition of the CDR3γ. The diversity of *TCRγ* CDR3 is generated not only by Vγ-Jγ rearrangement but also by the insertions of non-templated (N) and palindromic (P) nucleotides during the recombination process. N and P nucleotides as well as the exonuclease removals at the 3′ end of V segments and 5′ end of J segments were very common. For the potentially functional clones, the mean length of CDR3 was 38.69 ± 7.06 bp that encoded 4 to 36 (mean 12.90) AA (Fig. 8). Among them, more than 99.97 % of the unique CDR3γ sequences encoded 4 to 22 AA, which forms a typical Gaussian distribution. The lengths of CDR3γ formed by different V-J combinations showed marked differences (Fig. 6C, D). For Vγ segments, members of subgroup Vγ3 tended to form longer CDR3γ (mean 42.45 bp) than the other three subgroups (mean 34.13, 35.39 and 31.67 bp, for Vγ1, Vγ2 and Vγ4, respectively), probably because the germline CDR3 of Vγ3 (23 bp) is longer than those of Vγ1 (15 bp), Vγ2 (15 bp) and Vγ4 (13 bp). For the same reason, Jγ3 formed longer CDR3s (mean 39.76 bp) than Jγ1 (mean 37.84 bp) and Jγ2 (mean 37.93 bp). Furthermore, Vγ3 segments prefer to combine with Jγ3 (46.34 %) than the Vγ1 and Vγ2 segments (32.69 and 20.97 %) (see Additional file 8), also leading to form longer CDR3s.

## Discussion

The *TCRγ* locus is the smallest and least complex of the three conventional *TCR* loci and most considerably differ across species. By annotating the latest assembly of the red jungle fowl genome sequences, we found that chicken *TCRγ* locus spans about 100 kb, which is similar with opossum (90 kb), dromedary (105 kb), Chinese alligator (115 kb) and Rhesus monkey (120 kb), larger than dolphin (60 kb) and rabbit (70 kb) but smaller than human (160 kb), mouse (205 kb), sheep (250 kb, two loci), cat (260 kb), Atlantic salmon (270 kb, two loci), bovine (316 kb, two loci) and dog (460 kb) [21, 23–34]. As is reported in a previous study by Liu and colleagues [9], the immediately flanking the 3′ terminal of the *TCRγ* locus is a *LRRFIP2* (LRR binding FLII interacting protein 2) gene. However, the immediately flanking the 5′ terminal of this locus is an *AMPH* (amphiphysin) gene in the current genome sequence but a *PRKDC* (protein kinase DNA-activated catalytic polypeptide) gene in the previous study [9]. It is unclear which gene is correct, but a conserved *AMPH* gene was also identified at the same location flanking the *TCRγ* loci of many other species, such as human, mouse, opossum, rabbit, Chinese alligator, Rhesus monkey, dromedary, dolphin, cat and dog [21, 23, 25–27, 31–33, 40]. Although the chicken *TCRγ* locus is relatively smaller, it contains most (44) germline Vγ segments compared with species which have definite genomic maps of *TCRγ* loci [21–34]. Interestingly, the percentage of potentially functional Vγ genes in chicken is 63.64 % (28 of the 44 Vγ), which seems to be similar to human (6 of 14 Vγ, 42.86 %), cat (6 of 12 Vγ, 50 %), dog (8 of 16 Vγ, 50 %), duck (8 of 15 Vγ, 53.33 %), Atlantic salmon (7 of 11 Vγ, 63.64 %) and rabbit (8 of 11 Vγ, 72.73 %) and lower than sheep (11 of 13 Vγ, 84.62 %), dromedary (6 of 7 Vγ, 85.71 %), Chinese alligator (16 of 18 Vγ, 88.89 %), bovine (16 of 17 Vγ, 94.12 %), mouse (7 of 7 Vγ, 100 %) and opossum (9 of 9 Vγ,100 %) [21–31, 33, 34, 41]. Liu and colleagues previously identified 37 Vγ segments in chicken *TCRγ* locus, which could be divided into 11 subgroups [9]. Due to absence of the germline sequence of each Vγ in that study, we cannot establish the one-to-one correspondence between the Vγ segments identified in the present and previous studies. However, according to the mallard Vγ sequences used in phylogenetic tree in that study, we can speculate the possible corresponding relationship between the six Vγ subgroups identified now and the 11 Vγ subgroups identified previously (designated as preVγ1 to preVγ11) [9]. In detail, Vγ1 and Vγ5 probably corresponds to preVγ1; Vγ2 probably corresponds to preVγ3, 4, 5, 6, 7, 9, 10 and 11; Vγ3 probably corresponds to preVγ2; and Vγ4 and Vγ6 probably corresponds to preVγ8.

Unlike αβ T cells that require peripheral activation for differentiation into different effector cells, γδ T cells can be “developmentally programmed” in the thymus to generate different effector subsets. The thymic commitment to a γδ T cell fate at least in part requires the signal delivered by its γδ TCR [42]. In mice and humans, functionally distinct γδ T cell subsets can be defined by certain Vγ region (in mice) or Vδ region (in humans) that each subset expresses, [42–44]. During ontogeny of mice, waves of γδ T cell subsets possessing subset-characteristic Vγ (and sometimes pairing with certain Vδ) regions are successively generated in the thymus. Especially during fetal and early newborn life, several γδ T cell subsets containing invariant (or semi-invariant) γδ TCRs develop and acquire programmed effector functions in the thymus. After leaving the thymus, each γδ T cell subset migrates to distinct anatomical locations and performs particular functions [42–44]. Of note, in comparison with chicken, the germline repertoire of Vγ segments available for rearrangement is quite restricted in both humans and mice. In humans, only six to eight functional Vγ segments from two Vγ subgroups can be utilized in productively rearrangement [28, 41]. Although mice Vγ segments can be divided into five subgroups, except Vγ1 subgroup which has three functional Vγ segments, only one functional Vγ segment has been identified in each subgroup from Vγ2 to Vγ5 [29]. From this point view, the potentially combinational diversity of *TCRγ* is lower in humans and mice than in chickens, in which at least 21 Vγ segments from four Vγ subgroups are available for rearrangement in the present study. Furthermore, a preference of Vγ3.7, Vγ2.13, Vγ1.6 and Vγ1.3 segments is also a notable feature of the combinational diversity of *TCRγ* in chicken thymus. This feature was observed in all four 30-days-old individuals, indicating that this preference may be “developmentally programmed” in the thymus. Perhaps similarly to mice, certain chicken Vγ-specific TCRs (maybe also pairing with certain Vδ) might induce the acquisition of particular effector phenotypes at particular anatomical locations in the chicken, which is worthy of further study.

**Fig. 9:**
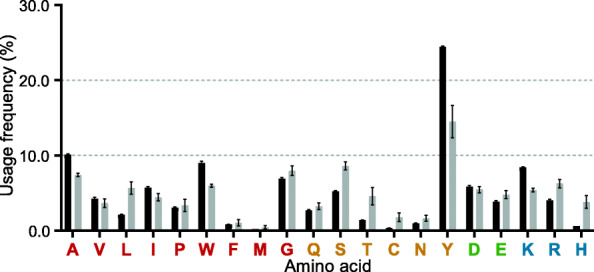
Composition of twenty essential amino acids in the CDR3γ. Hydrophobic, neutral-hydrophilic, acidic and basic amino acids are depicted below the horizontal axis in red, yellow, green and blue, respectively. Black and gray columns represent the mean frequency of certain amino acid calculated from normal CDR3γ (4 to 22 AA) and ultralong CDR3γ (23 to 36 AA), respectively. The mean frequency was calculated from four individuals, and the error bars show standard deviations

The length distribution of the CDR3 has been used as a metric in assessments of the possible range of binding paratope generated by a given TCR type. By either traditional cloning and sequencing (hereafter called low-throughput sequencing, LTS) or HTS, the length distribution of the CDR3γ (AA numbers) has been analyzed in the following species, including humans: 4 to 15 (mean 10.2) AA by LTS and 6 to 20 AA by HTS [45–48]; mice: 7 to 14 (mean 11.8) AA by LTS and 6 to 16 AA by HTS [45, 49]; ducks: 5 to 19 (mean 11.0) AA by LTS [22]; platypus (*Ornithorhynchus anatinus*): 9 to 15 AA by LTS [38]; Chinese alligator: 4 to 17 (mean 11.3) AA by LTS [23]; Florida manatee (*Trichechus manatus latirostris*): 5 to 21 (mean 10.6) AA by HTS [50]; nurse shark (*Ginglymostoma cirratum*): 9 to 15 (mean 12.1) AA by LTS [51] and Japanese flounder (*Paralichthys olivaceus*): 8 to 13 (mean 11.5) AA by LTS [52]. In this study, we obtained a more accurate length distribution of the chicken CDR3γ based on HTS. The vast majority of the chicken CDR3γ sequences encoded 4 to 22 with mean 12.90 AA, which exhibits a wider length distribution and/or a longer mean length than the data from most other species mentioned above, indicating that this vast length variability would markedly increase the sequence/structural diversity of chicken *TCRγ* chains, which could presumably affect pairing with the TCRδ chain and downstream signaling or effector functions. By HTS, we also found 138 “ultralong CDR3γ (23 to 36 AA), though they just account for less than 0.025 % of the total CDR3γ sequences. The AA composition of the normal CDR3γ (4 to 22 AA) and ultralong CDR3γ were analyzed separately (Fig. 9). Compared with normal CDR3γ, ultralong CDR3γ tended to use less hydrophobic AA (42.20 % vs. 39.96 %), but more hydrophilic AA (57.80 % vs. 60.04 %). The tyrosine content of ultralong CDR3γ (14.50 %) was significantly lower than that of normal CDR3γ (24.46 %), but the usage of other neutral and hydrophilic AA (including serine, threonine, asparagine and glutamine) in ultralong CDR3γ was higher than that in normal CDR3γ (Fig. 9). These results indicated that the ultralong CDR3γ might form unusual architecture for antigen binding. We also found that the cysteine residue was strongly preferred in ultralong CDR3γ than normal CDR3γ (1.76 % vs. 0.33 %), suggesting that the ultralong CDR3γ might use interloop disulfide bond to maintain the structural stability of the long CDR3γ loop.

Finally, earlier studies of chicken T cell development indicated that the chicken thymus is colonized with thymocyte precursors in three discrete waves during embryogenesis [53]. The γδ T cells produced from each wave exit rapidly from the thymus without undergoing clonal expansion and colonize peripheral organs such as spleen and intestine [39]. By using semiquantitative PCR and LTS of the *TCRγ* transcripts, subsequent studies showed that although the precursors of each wave rearranged all three subgroups (subgroup Vγ1 to Vγ3) identified then, each wave displayed a variable repertoire, indicating that the repertoire diversities of *TCRγ* in the thymus are likely to change with ontogeny of chicken [54]. In this study, we only focused on the *TCRγ* repertoire acquired from the thymus of 30-days-old hens, and future research can use HTS to survey the repertoire diversities of *TCRγ* in both thymus and peripheral lymphoid tissues during the ontogeny of chicken, which may contribute to discover the similarities and differences in development of the gd T cells between birds and mammals or between “γδ-low” and “γδ-high” species.

## Conclusions

In this study, we analyzed the chicken thymus *TCRγ* repertoire based on the germline Vγ and Jγ segments identified in the latest assembly of the red jungle fowl genome sequences (GRCg6a). The notable features of chicken thymus *TCRγ* repertoire include a biased usage of several Vγ segments and Vγ-Jγ pairs, as well as a wider length distribution of the CDR3γ. We hope that our characterization of chicken *TCRγ* repertoire can widen the understanding of adaptive immunology in birds and benefit future research on adaptive immune responses of chicken in health and disease.

## Supplementary Information

**Additional file 1.** The accession numbers of Vγ segments used in phylogenetic analysis.

**Additional file 2.** Primers used in this study.

**Additional file 3.** Phylogenetic analysis of members from Vγ2 subgroup. The phylogenetic tree was constructed using the Neighbor Joining method in MEGA X with nucleotide sequences corresponding to FR1 through FR3. Bootstrap percentage values based on 1000 replicates are shown at the interior branch nodes.

**Additional file 4.** Detailed information of the germline Vγ, Jγ, and Cγ gene segments retrieved from genomic sequences of red jungle fowl (GRCg6a).

**Additional file 5.** Nucleotide sequence similarities between homology units by pairwise alignment.

**Additional file 6.** Phylogenetic analysis of Jγ segments from chicken and duck. The phylogenetic tree was constructed using the Maximum likelihood method in MEGA X with nucleotide sequences of Jγ segments. Bootstrap percentage values based on 1000 replicates are shown at the interior branch nodes. Chicken Jγ segments are shown in bold.

**Additional file 7.** Usage frequencies of all possible Vγ-Jγ pairs in each individual. The vertical axis represents all potentially functional Vγ segments and the horizontal axis represents three Jγ segments. The color depth is proportional to the usage frequency of a certain Vγ-Jγ pair.

**Additional file 8. **Usage frequencies of three Jγ segments paired with different Vγ subgroups.

## Data Availability

The raw sequence reads generated from HTS were submitted to Sequence Read Archive (SRA) database (accession number: PRJNA714701,https://www.ncbi.nlm.nih.gov/sra/PRJNA714701).

## Abbreviations

TCR: T cell receptor
RACE: Rapid Amplification of cDNA Ends
HTS: High-throughput sequencing
CDR3: Complementarity-determining region 3
CD3: Cluster of differentiation 3
MHC: Major histocompatibility complex
IMGT: The international ImMunoGeneTics information system
BAC: Bacterial artificial chromosome
SMRT: Single molecule real time sequencing technology
RSS: Recombination signal sequence
ORF: Open reading framework
Phe: Phenylalanine
Gly: Glycine
Tyr: Tyrosine
His: Histidine
CYS: Cysteine
TRP: Tryptophan
FR: Framework region
qRT-PCR: Quantitative real-time polymerase chain reaction
AA: Amino acids
3′ UTR: 3′ untranslated region
