# Correction to: Characterization of the chicken T cell receptor γ repertoire by high-throughput sequencing

**DOI:** 10.1186/s12864-021-08136-6

**Published:** 2021-11-29

**Authors:** Tongtong Zhang, Qian Li, Xiaoqing Li, Li Kang, Yunliang Jiang, Yi Sun

**Affiliations:** Control and Prevention, Shandong Agricultural University, 61 Daizong Street, Shandong Province 271018 Taian City, People’s Republic of China

**Correction to: BMC Genomics 22, 683 (2021)**

**https://doi.org/10.1186/s12864-021-07975-7**

Following publication of the original article [1], an error was identified in the Methods section.

It currently reads:

In “Library preparation, HTS and data analysis” in the Methods section, the description “Overlapping paired-end reads from the 5′ RACE-based library were merged with FLASH [20], and these merged sequences were aligned to the germline Vγ and Jγ segments identified above using a local BLAST program (version 2.2.30) and each sequence was assigned an optimal germline Vγ and Jγ segments” is wrong. Actually, the atuhors used the 3′ end reads which contain the Cγ-specific reverse primer for 5′ RACE and 250 bp in length to analyze the optimal germline Vγ and Jγ segments that participated in Vγ-Jγ rearrangement. This error does not influence any conclusion of this paper.

It should read:

In “Library preparation, HTS and data analysis” in the Methods section, use “The 3′ end reads which contain the Cγ-specific reverse primer for 5′ RACE and 250 bp in length were aligned to the germline Vγ and Jγ segments identified above using a local BLAST program (version 2.2.30) and each sequence was assigned an optimal germline Vγ and Jγ segments.”

All the changes requested are implemented in this correction and the original article [1] has been corrected.
