# Supplementary figures and images for: Characterization of the chicken T cell receptor γ repertoire by high-throughput sequencing
Source: BMC Genomics. 2021 Sep 21;22:683. doi: 10.1186/s12864-021-07975-7 (PMC8456604; doi:10.1186/s12864-021-07975-7)

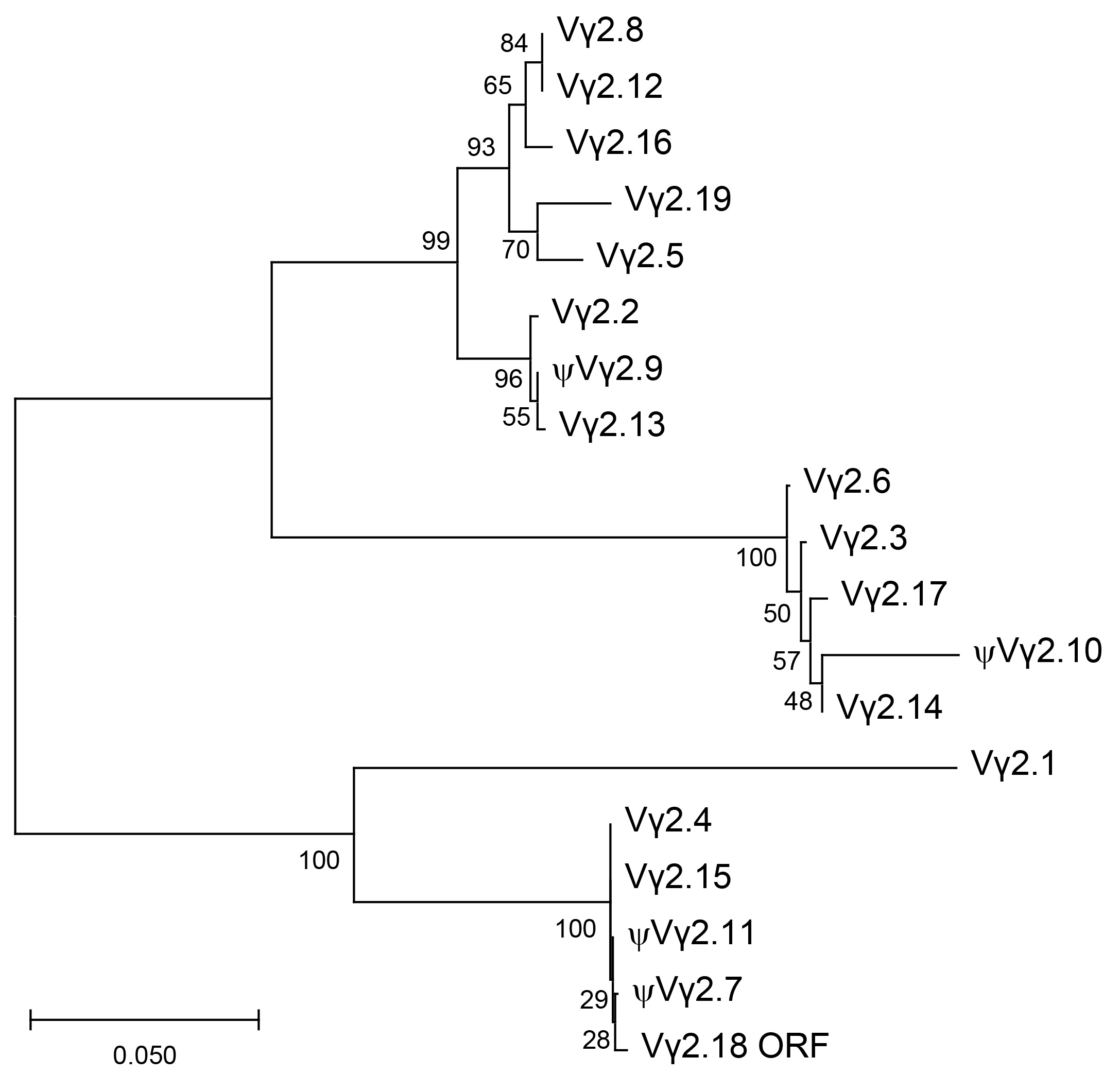

Supplement: Supplementary file 3 — Additional file 3. Phylogenetic analysis of members from Vγ2 subgroup. The phylogenetic tree was constructed using the Neighbor Joining method in MEGA X with nucleotide sequences corresponding to FR1 through FR3. Bootstrap percentage values based on 1000 replicates are shown at the interior branch nodes. [file 12864_2021_7975_MOESM3_ESM.jpg]

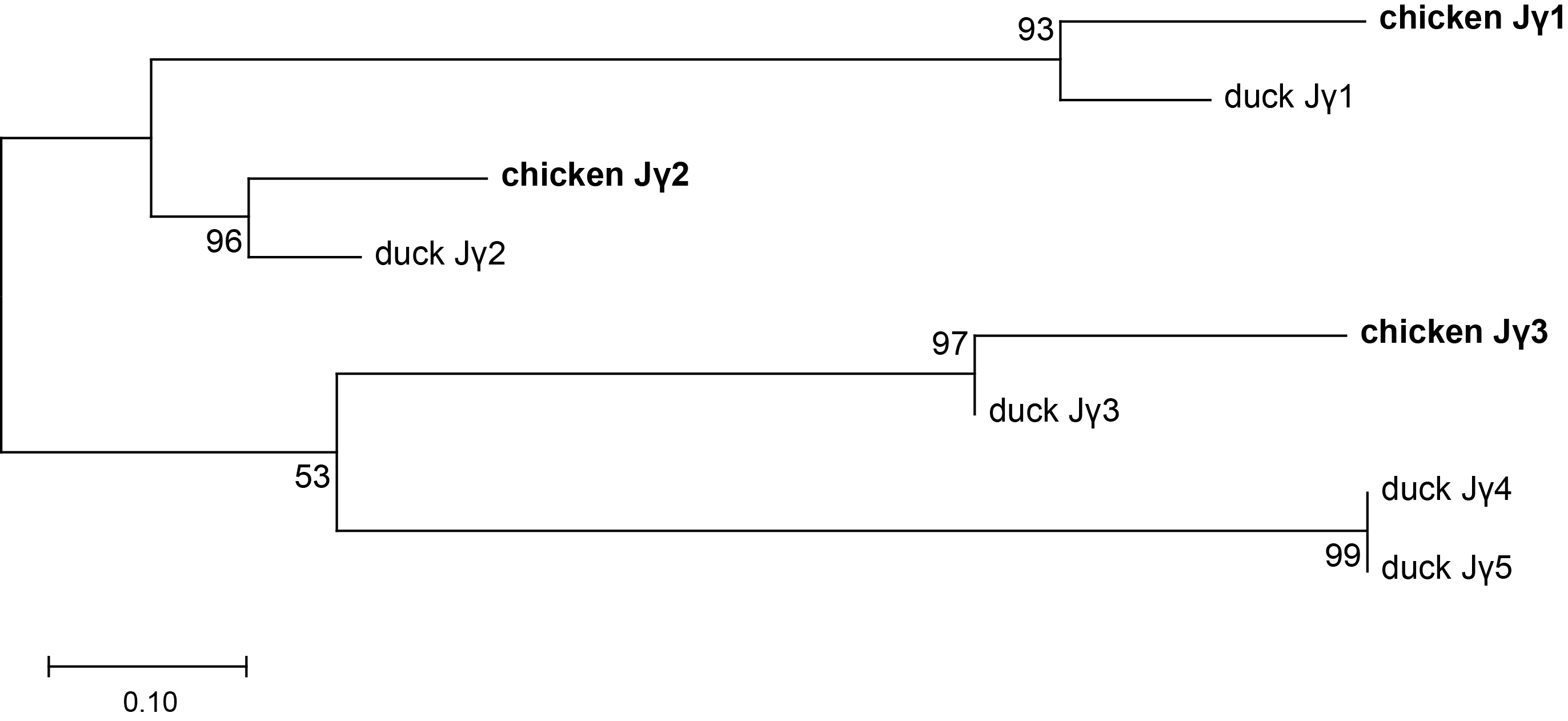

Supplement: Supplementary file 6 — Additional file 6. Phylogenetic analysis of Jγ segments from chicken and duck. The phylogenetic tree was constructed using the Maximum likelihood method in MEGA X with nucleotide sequences of Jγ segments. Bootstrap percentage values based on 1000 replicates are shown at the interior branch nodes. Chicken Jγ segments are shown in bold. [file 12864_2021_7975_MOESM6_ESM.jpg]

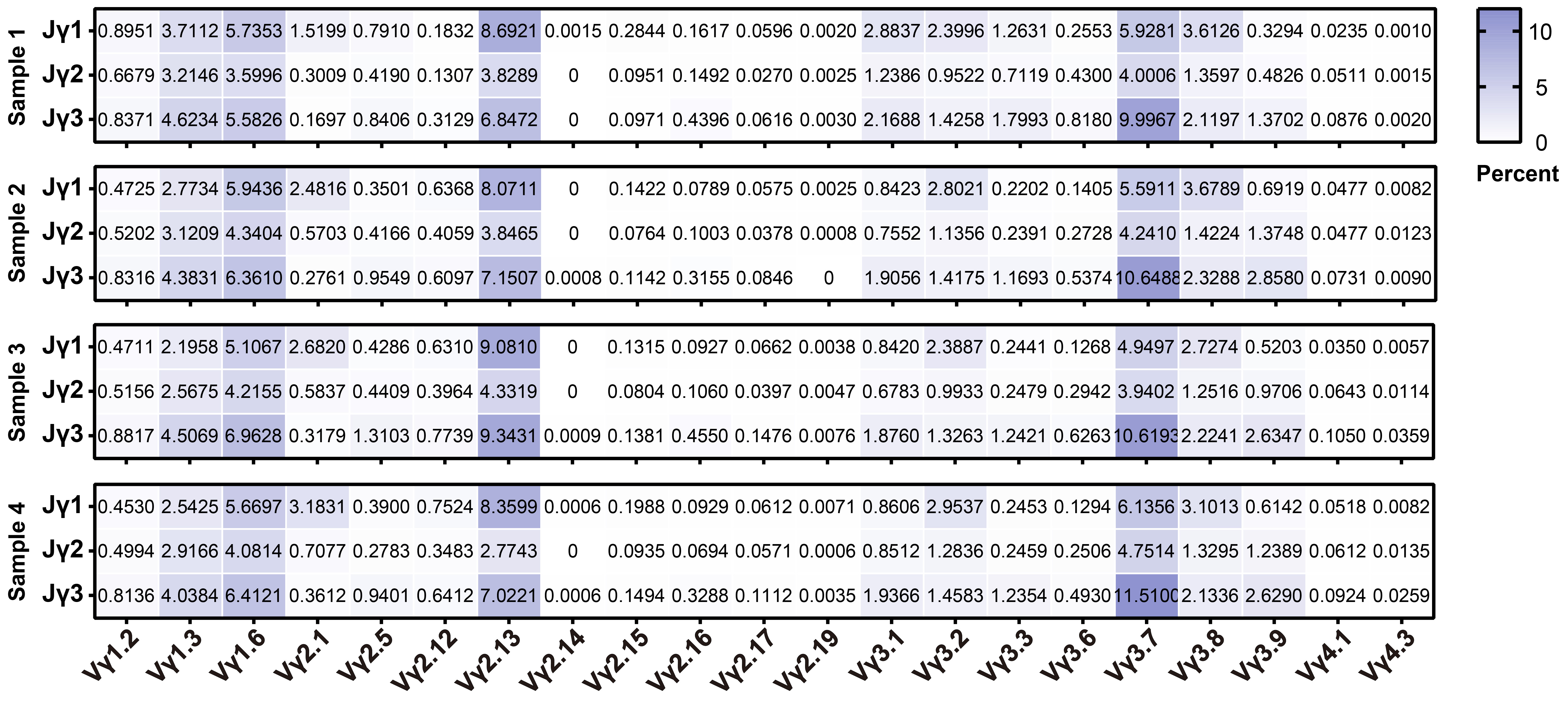

Supplement: Supplementary file 7 — Additional file 7. Usage frequencies of all possible Vγ-Jγ pairs in each individual. The vertical axis represents all potentially functional Vγ segments and the horizontal axis represents three Jγ segments. The color depth is proportional to the usage frequency of a certain Vγ-Jγ pair. [file 12864_2021_7975_MOESM7_ESM.jpg]

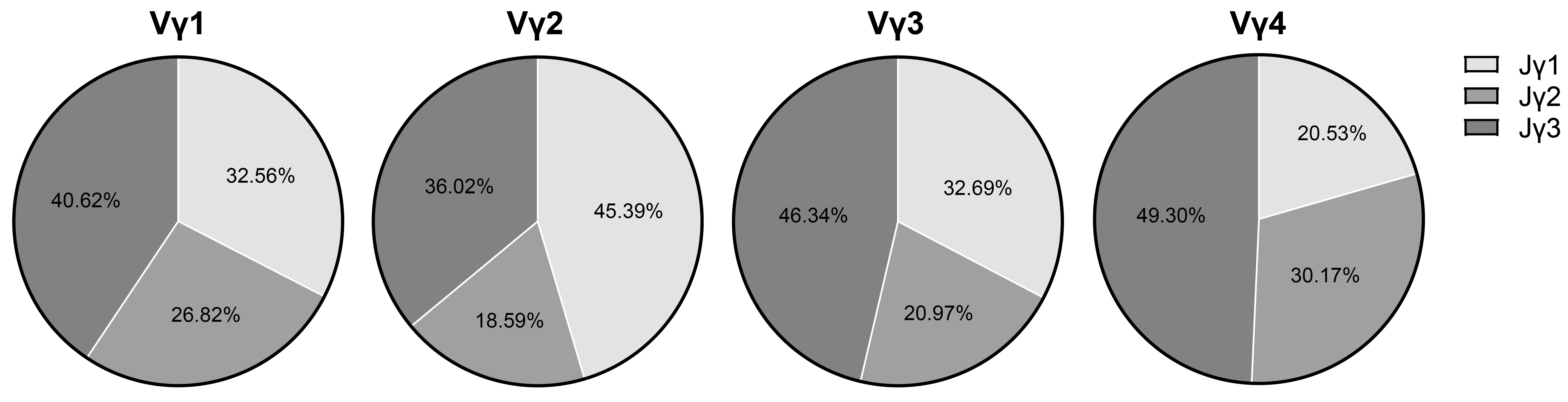

Supplement: Supplementary file 8 — Additional file 8. Usage frequencies of three Jγ segments paired with different Vγ subgroups. [file 12864_2021_7975_MOESM8_ESM.jpg]
